# On the evidence of helico-spiralling recirculation within coherent cores of eddies using Lagrangian approach

**DOI:** 10.1038/s41598-024-61744-6

**Published:** 2024-05-14

**Authors:** Rahul Deogharia, Hitesh Gupta, Sourav Sil, Avijit Gangopadhyay, Abhijit Shee

**Affiliations:** 1 Ocean Analysis and Modeling Laboratory, School of Earth, Ocean and Climate Sciences, Indian Institute of Technology Bhubaneswar, Khordha, Odisha 752050 India; 2 School for Marine Science and Technology, University of Massachusetts, Dartmouth, 02747 MA USA; 3 Centre for Atmospheric and Oceanic Sciences, Indian Institute of Science, Bengaluru, Karnataka 560012 India

**Keywords:** Coherent eddies, Lagrangian Coherent Structures (LCSs), Lagrangian Averaged Vorticity Deviation (LAVD), Upwelling, Downwelling, Vortex Rossby Waves (VRWs), Physical oceanography, Physical oceanography

## Abstract

Oceanic eddies exhibit remarkable coherence and longevity compared to other transient features in the surrounding flow. They possess the ability to transport properties over extensive distances while maintaining their material identity intact. The Lagrangian Coherent Structure (LCS) framework has proven effective in capturing these coherent eddies, where they display a solid-body-like rotation. Although various LCS approaches have been employed to investigate different facets of coherent eddies, a comprehensive understanding of their three-dimensional structures and internal dynamics remains elusive. This study aims to advance our comprehension of coherent eddies’ structural characteristics and delve into the precise nature of their internal dynamics by utilizing the Lagrangian Averaged Vorticity Deviation approach. Two eddies, one cyclonic and the other anti-cyclonic, were chosen from a high-resolution simulation carried out in the Bay of Bengal using the Regional Ocean Modeling System (ROMS). The findings unveil that these eddies have three-dimensional coherent cores resembling gently tapered cones that are broader at the surface and gradually narrow towards the bottom. Intriguingly, the dynamically coherent core of these eddies exhibits simultaneous upwelling and downwelling while maintaining their volumes during advection due to persistent material coherence. The three-dimensional trajectories followed by the fluid parcels inside the coherent core are helical. Their two-dimensional horizontal projections show alternating spiral bands of upwelling and downwelling which are the manifestations of Vortex Rossby Waves. These observations lead to a conceptual framework of a three-dimensional helico-spiralling recirculation pattern within the coherent cores of eddies.

## Introduction

Eddies are one of the predominant features of the ocean circulation which often result from instabilities of persistent meandering currents^1^, and in turn limit the strength of these persistent currents^2^. They are also formed due to mixing between circulating water masses, wind-driven upwelling or downwelling in the open ocean, and flow interactions with coasts and topography^3^. Their horizontal scales range from tens to hundreds of kilometers with dominant temporal scales between days to months. Their primary signature is a closed vorticity contour which remain intact for most of their lifetime. Of all the oceanic circulations, mesoscale eddy fields account for the peak in the kinetic energy spectrum^2^ at sub-inertial frequencies^4^. Their initial energy generally cascades down to eddies of smaller and smaller scales, till eventually, viscous diffusion takes over^5,6^. Eddies play a crucial role in the transport of mass, energy, and tracers across the global oceans, in quantities comparable to that of the large-scale wind- and thermohaline-driven circulation, thereby modulating the global climate^7^. Oceanic eddies also play a crucial role in modulating air-sea interactions. Studies have reported that the passage of tropical cyclones over warm-core anti-cyclonic eddies leads to the intensification of the cyclones^8–10^. Recently, Gupta et al.^11^ showed the importance of such eddies in transporting marine heat waves in the BoB. Furthermore, not only does atmospheric deep convection occur over regions of mesoscale anti-cyclonic eddies during active monsoon^12^, these oceanic mesoscale features also change the local association between convective rainfall and sea-surface temperature^13^. Despite their importance in ocean circulation and climate dynamics, our understanding of oceanic eddies is limited as their origin lies in turbulence. In the absence of an exact theory of turbulence, only numerical modeling has been the primary tool for studying different aspects of eddy dynamics^14^. Large-scale climate and General Circulation Models (GCM) either parameterize or can partially resolve the effects of eddies. The submesocale and micro-scale eddies cannot be resolved by GCMs and hence capturing the effects of such eddies need better parameterization schemes^2^, which requires establishing objective definitions for these entities and understanding their interior dynamics.

Significant number of studies have focused on understanding the eddy interior and peripheral dynamics using both Eulerian and Lagrangian frameworks. Koszalka *et al.*^15^, Nardelli^16^, and Zhong *et al.*^17^ have tried to analyze the vertical dynamics of the mesoscale coherent anti-cyclonic and cyclonic eddies, however, no objective definition of coherence was utilized. In the process, they discovered that fine-scale vertical velocity structures are indicative of Vortex Rossby Waves (VRWs), which are radially outward propagating azimuthal waves of potential vorticity^18–21^ or vertical velocities^15,16^. It has also been found that the VRWs have smaller angular speeds than the fluid parcels, and hence the latter cross alternating bands of positive and negative vertical velocities associated with the VRWs, resulting in fluctuating vertical displacement of the parcels^16,18^. A detailed theory and characterization of VRWs have been proposed, wherein different aspects of VRWs have been investigated^18–20^. It has been shown that VRWs arise from ageostrophy^15^ which results in strong horizontal convergence and vertical velocities in the submesoscale realm^22,23^. Moreover, it was pointed out by Koszalka *et al.*^15^, that there may not exist a trivial relationship between the vertical velocity and vorticity structures due to the dominance of ageostrophic effects. Also, Nardelli^16^ has pointed out that conclusive characterization of VRWs based on theory (as given by McWilliams *et al.*^19^) may not be possible as the assumptions of scale separation between the waves and the vortex, and isolation of the vortex from the mean flow may not always hold. Zhong et al.^17^ explored the impact of model resolution on the ability to capture small-scale vertical velocity structures. It was found that Lagrangian particle evolution shows spiralling bands of positive and negative vertical displacements. Even though this spiralling was evident in models of different resolutions, however, the vertical displacement was more vigorous when the spatial resolution was increased (from 10 km to 5 km to 1 km). Zhang et al. reported that the spiral bands are fine structures of mesoscale eddies that can greatly enhance the surface chlorophyll concentration along band structures, which can be explained as biogeochemical footprints of the vertical motions induced by VRWs^24^. Though the aforementioned literature studied eddies (particularly coherent in nature), the internal eddy structures were not rigorously identified.

Two approaches are generally used to characterize the internal eddy structure—Eulerian based diagnostics^25–30^ and Lagrangian based methods^31,32^. The Eulerian methods have been shown to lack objectivity (frame-invariance)^33,34^ and suffer from the inability to coherently track the evolution of fluid parcels in time-dependent flows^34^. However, the eddies detected using Eulerian diagnostics have a smaller materially coherent core inside^35,36^. These coherent vortices are concentrated patches of vorticity that last longer than other structures in the ambient flow thereby maintaining their material identity^32,37–39^. These materially coherent vortices show solid-body-like rotation, wherein, all the water parcels have the similar average angular velocity rotating about some center of the coherent core^40,41^. These coherent cores can be identified using Lagrangian methods as elliptic Lagrangian Coherent Structures (LCSs)^34,42–45^, which by definition, are impermeable to advective fluxes and can transport passive tracers over large distances^32,38,46^.

Significant research has been dedicated to examining the characteristics and behavior of coherent eddies. Some of the early studies on different vortex processes using the ideas of Finite Size Lyapunov Exponent (FSLE) based LCSs include the study of global chaotic mixing on isentropic surfaces in the troposphere using general circulation model simulations^47^, numerical 2D-turbulence simulations^48^, and analysis of mixing structures in the Mediterranean using a primitive equation circulation model^49^. The quasi-three-dimensional structure of a cyclonic eddy was reconstructed from the attracting and repelling LCSs, computed from the ridges in the FSLE fields for the Benguela upwelling region^43^; the FSLE fields quantify the rate of growth of finite-size material perturbations^34,50^. While the FSLE method does not show the material coherence of an eddy, it can uncover the geometry and transport processes across the eddy. Liu *et al.*^40^ carried out an extensive comparison of the sizes, lifespans, and mass transport between the two-dimensional Sea Surface Height (SSH) based Eulerian eddies and their coherent counterparts obtained using the Lagrangian Averaged Vorticity Deviation (LAVD)^32^ approach. They found that these coherent counterparts form the core of the SSH-based eddies, and have an average size about half of that of the SSH-based eddies. Moreover, Abernathey & Haller^35^ reported that Lagrangian coherent eddies have a lifespan shorter than their SSH-based counterparts.

It has also been suggested that the coherent core of an eddy is surrounded by a semi-coherent region^51^ that constantly interacts with the surrounding flow. As a result, it intermittently loses and regains its material coherence with time. The coherent cores together with their surrounding semi-coherent regions are identified as the whole eddy system by the Eulerian methods^41,51^. Xia et al.^52^ have studied the coherent mass transport due to coherent eddies in the global oceans and have found that it is one order of magnitude smaller than Eulerian estimates. This study also explored the 3D structures of LAVD-based coherent eddies and Vector-Geometry^26^ based Eulerian eddies and found that the coherent eddies retain their material identity while the Eulerian eddies quickly filament out, losing the tracers inside to the ambient flow. The anti-cyclonic coherent eddy showed compactness which was attributed to the downwelling and convergence of the flow field, however, its detailed dynamics were not explored. Though we have made significant progress towards understanding the characteristics and behavior of coherent eddies as a core of Eulerian-based eddies, their detailed characterization in three dimensions is still lacking. Additionally, the dynamics happening in the interior of the coherent core of eddies need to be explored in greater depth. Such a dynamical understanding also has to be reconciled with the processes behind eddy-induced upwelling and downwelling. The present study aims to shed some light on these questions through a detailed Lagrangian analysis of coherent cores of eddies.

In this study, we have identified the three-dimensional coherent cores associated with oceanic eddies, both Anti-Cyclonic (ACE) and Cyclonic (CE), using the Lagrangian Averaged Vorticity Deviation (LAVD) approach^32^ on the ocean currents simulated at 5km horizontal resolution for the Western Bay of Bengal (WBoB) domain using the Regional Ocean Modeling System (ROMS)^53,54^. The interior dynamics of these coherent eddies have been studied in detail through Lagrangian particle advection, in which a set of initially seeded particles are followed as passive tracers in space and time using the three-dimensional flow velocity field. Finally, a conceptual circulation scheme for the interior of these coherent eddies has been proposed that reconciles both the material nature of these eddies and the upwelling/downwelling induced by them.

## Results

### Model validation

**Figure 1:**
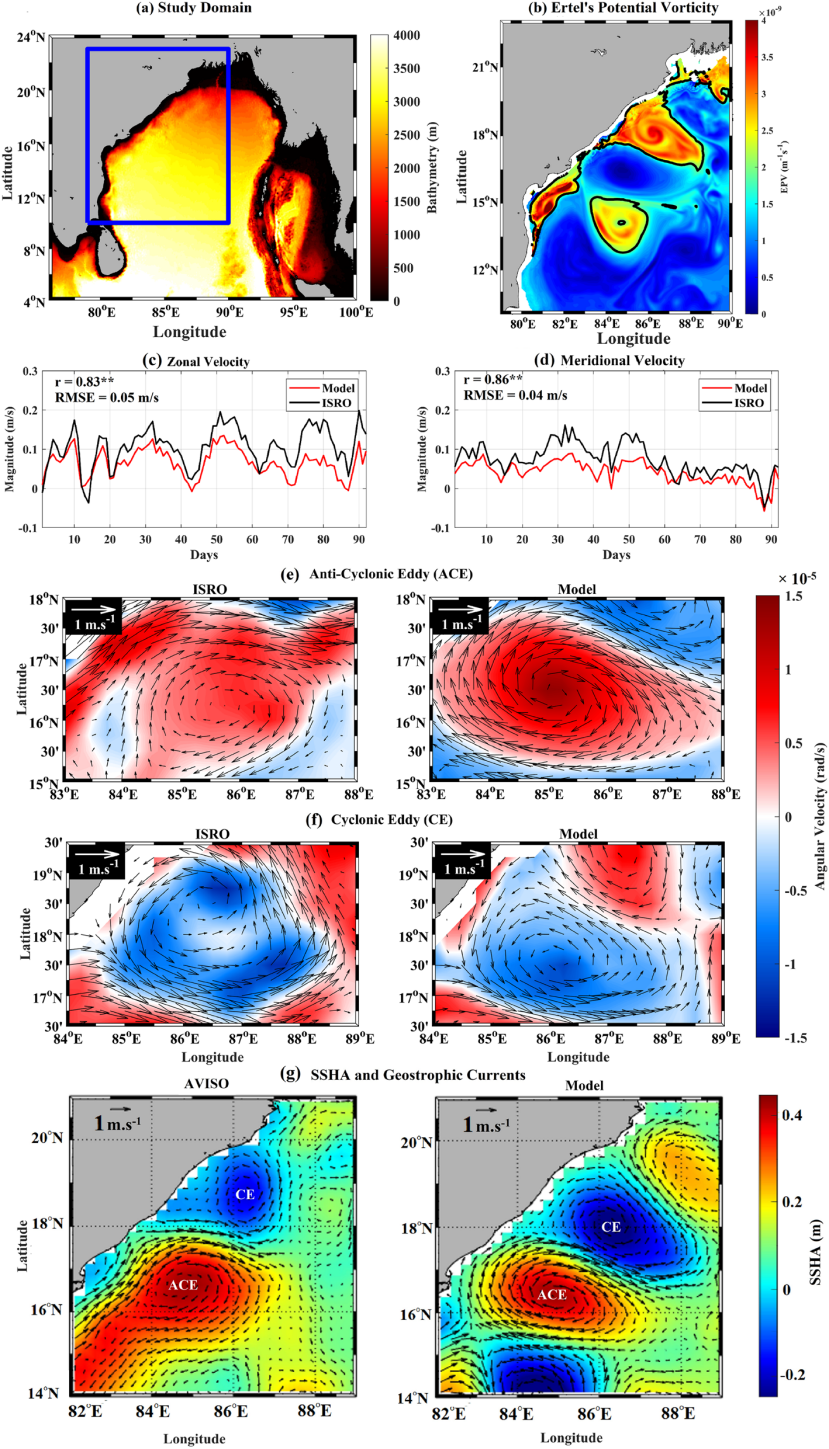
(**a**) Map of the Bay of Bengal with the nested WBoB ROMS domain marked by solid blue box and the colormap representing bathymetry, (**b**) Map of EPV for 08th April, 2017, with the isoline of EPV = $2 \times 10^{-9} m^{-1}s^{-1}$ (solid black), (**c**) timeseries of the area-averaged zonal component and (**d**) meridional component ROMS simulated surface currents (solid red line) and ISRO currents (solid black line) from 01st March–31st May, 2017. Panels (**e**) and (**f**) compares the map of the ISRO and model surface currents (averaged over the time periods as mentioned in Table 1) overlaid on the angular velocity ($\omega = 1/2 \nabla \times {\textbf{v}}$; where ${\textbf{v}}$ is the velocity field) contours, for the Anti-Cyclonic Eddy (ACE) and Cyclonic Eddy (CE) respectively. Panel (**g**) compares the model generated SSH and geostrophic currents against AVISO data for 08th April, 2017.

The region of study is the WBoB (79° E–90° E and 10° N–23° N; Figure 1) where the Western Boundary Current (WBC) is prominent during the spring season^55^. The enhanced instability in this current results in a region of high eddy kinetic energy^56^. The WBC separates from the coast at around 18° N and forms a strong eastward-jet following an isoline of Ertel’s Potential Vorticity (EPV) and flows between two pools of high EPV; see Fig. 1b. There exists a cyclonic eddy (anti-cyclonic eddy) to the north (south) of this eastward-jet^55^ (see Fig. 1b,g).

The area-average of the ROMS simulated surface-currents (see Methods for details on model setup) were validated against the surface currents provided by the Indian Space Research Organisation (ISRO) which assimilated multiple satellite altimeter data over a larger Indian Ocean region^57^. It can be seen that the model simulated currents closely follow the ISRO currents with a small but consistent underestimation (Fig. 1c–d). The correlation coefficient (*r*) and the Root Mean Squared Error (RMSE) between the components (zonal and meridional) of the ROMS and ISRO surface currents are indicated in the same figures. Moreover, it can be seen that the model-generated surface currents capture the eddies well, which can also be observed on the ISRO surface currents (Fig. 1e–f). However, the angular velocity (i.e., half of vorticity) extremum in the cyclonic eddy is relatively off to a side from the center of the circulation pattern as compared to the anti-cyclonic eddy. Finally, the model generated SSHA and the geostrophic currents obtained from it also show the eddies that can also be seen from the AVISO data (Fig. 1g). These two well-validated eddies are the subject of this study.

**Figure 2:**
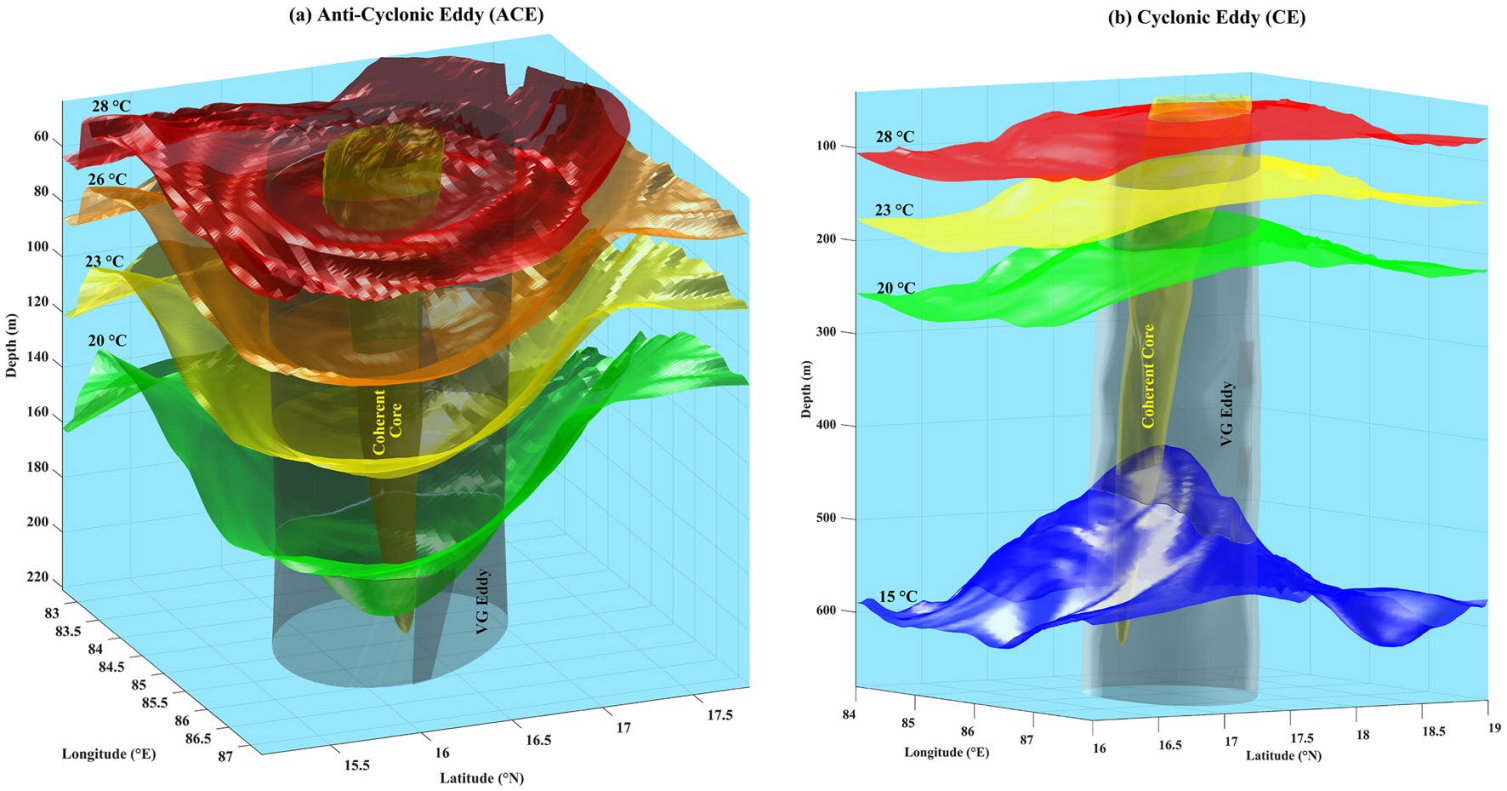
(**a**) The three-dimensional structure of the coherent core (yellowish-green; translucent) and the Vector-Geometry (VG) based structure of the Anti-Cyclonic Eddy (ACE) (black; translucent). (**b**) same as (**a**) but for Cyclonic Eddy (CE). The different colored surfaces are temperature isosurfaces for 28 °C (red), 26 °C (orange), 23 °C (yellow), 20 °C (green), and 15 °C (blue).

### Identification of Eulerian-based eddies and their coherent cores

The structures of the Eulerian-based eddies were determined using the Vector-Geometry (VG) based method^26^ (see Methods) applied to two-dimensional horizontal sections of currents separated by 5 m intervals vertically (starting from 1 m depth) obtained from the ROMS model (5 km horizontal resolution). The three-dimensional structures were then reconstructed from these two-dimensional eddy boundaries at different depths using linear interpolation.

The three-dimensional coherent cores, associated with these VG-based eddies, were identified by first calculating the LAVD (defined as the trajectory integral of the normed deviation of the vorticity from its spatial mean; see Methods) fields and then by identifying the outermost convex isosurfaces of these fields^32^. The LAVD computation requires a 3-D gridded velocity information at every time-step from start to end of the advection period and beyond to test the coherence of the Lagrangian vortex. In our study, these LAVD computations were carried out for three different regular grids with horizontal resolutions of 5 km, 3 km, and 1 km, corresponding temporal resolutions of 1 day, 1/10 days, and 1/15 days, and a common vertical resolution of 5m, respectively. The velocity fields for LAVD computation were obtained for each of these LAVD computational grids via appropriate spatio-temporal interpolation from the ROMS simulated currents at 5 km horizontal resolution. The Lagrangian coherent eddy structure as identified from the 1 km and the 3km resolution computations are very similar, but, are smaller and more compact compared to the 5 km resolution computations (Supplementary Figure 1). Considering increased compactness, better coherence, and reduced filamentation, the analyses using the 1km resolution computations are presented here.

The eddies identified using the VG-based method were found to be much larger than their LAVD-based coherent core (Fig. 2). However, unlike their coherent cores, the VG-based eddies do not show any closure towards the bottom. The coherent core of the anti-cyclonic eddy is well aligned with its VG-based counterpart (Fig. 2a). However, in the case of the cyclonic eddy, it was observed that the axes of the VG-based eddy and its coherent core are misaligned (Fig. 2b) due to a skewed spatial distribution of vorticity (Fig. 1f). Moreover, the VG-based eddies do capture the signatures of upwelling and downwelling (for the anti-cyclonic eddy and cyclonic eddy respectively) as can be seen from the isotherm surfaces. However, these signatures are much less pronounced inside their coherent cores owing to their comparatively smaller sizes^40^. It can be observed that the isotherm surfaces move downward (upward) for the anti-cyclonic eddy (cyclonic eddy) around the coherent cores following the right-hand thumb rule. A potential reason behind this could be that these coherent cores, given their solid-body-like rotation^40,41^, act like screws that would go down or up for clockwise or anti-clockwise rotation respectively, shearing the nearby isothermal surfaces in the same direction.

### Characteristics of the coherent cores

**Figure 3:**
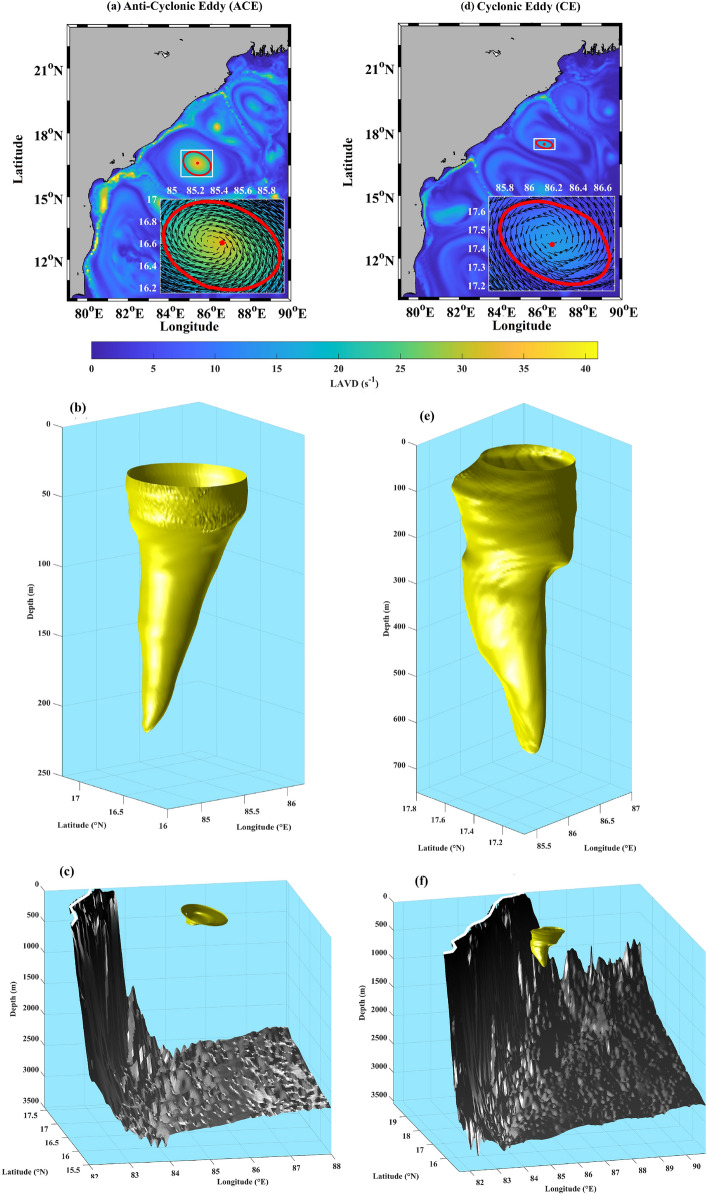
(**a**) Map of LAVD field at 30 m overlaid with outermost contour of LAVD corresponding to the Anti-Cyclonic Eddy (ACE), (**b**) The isolated three-dimensional structure of the ACE for a detailed view, (**c**) The three-dimensional structure of the ACE (yellow) and the bathymetry (black) with the solid white line representing the coastline. (**d**), (**e**), and (**f**) are same as (**a**), (**b**), and (**c**), respectively but for Cyclonic Eddy (CE). The insets in panels (**a**) and (**d**) show a zoomed-in version of the ACE and CE, respectively, overlaid with the surface-currents for the first day of integration period (Table 1).

The calculated LAVD fields overlaid with the identified coherent cores associated with the anti-cyclonic eddy and cyclonic eddy show a good correspondence with the surface-currents (see the insets in Fig. 3a and d). Figure 3b and e show the three-dimensional structures of these identified coherent eddy cores. The first thing to note is that the cyclonic eddy (~ 620 m) extends much deeper than the anti-cyclonic eddy (~ 210 m). Here, the anti-cyclonic eddy is formed partly by the separating WBC whose vertical extent is limited to the upper 200 to 400 m^55^. Also, the upper part of the surface of the anti-cyclonic eddy shows small-scale ripples (up to about 75 m) as compared to the surface of the cyclonic eddy which is relatively smoother. This may be due to the deviation of the actual eddy structure from a perfect convex hull as encoded by the *convexity deficiency* parameter^32,44^. Moreover, the cyclonic eddy shows distinct bulges and depressions resulting in an overall distorted appearance. Both the eddies are far from both the bottom topography and the coastline to be significantly impacted by them (Fig. 3c and f). It can also be observed that coherent eddy structures are vertically tilted, which was also reported by Li et al.^58,59^. Moreover, there is a distinct asymmetry between the LAVD strength of the anti-cyclonic eddy and the cyclonic eddy. This results in different Lagrangian advection behavior of these eddies as discussed below.

### Lagrangian particle advection

To study the dynamical behavior of the eddy boundaries and their interiors, a complete three-dimensional Lagrangian advection of the boundaries and particles seeded at different vertical levels inside the eddies were performed. The period of advection was kept the same as the integration period with a time-step of 1/15 days (Table 1). The advection of the boundaries, with their corresponding surface structures and currents, shows that the small-scale ripples observed on the surface of the anti-cyclonic eddy eventually grow into filaments upon advection (Supplementary Videos 1 and 2, Supplementary Text 1). Such filamentation has been attributed to the loss of material coherence through the exchange of material with the ambient flow^44^.

**Figure 4:**
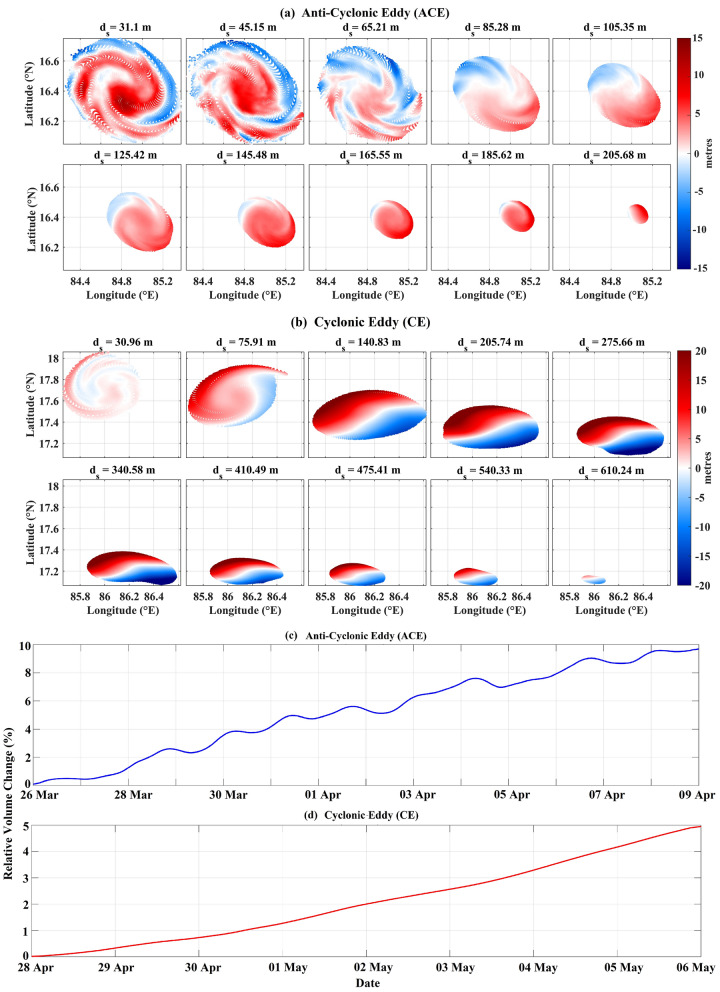
(**a**) The difference between the vertical positions of the particles at the last and first day of the advection (see Table 1) for the Anti-Cyclonic Eddy (ACE). The colorbar shows this difference in meters. (**b**) Same as (**a**) but for the Cyclonic Eddy (CE). The top of the figures in panels (**a**) and (**b**) show the starting depth ($d_{s}$) of the particles before advection. Timeseries of relative volume change plotted against the day of advection for (**c**) the ACE and (**d**) the CE.

**Figure 5:**
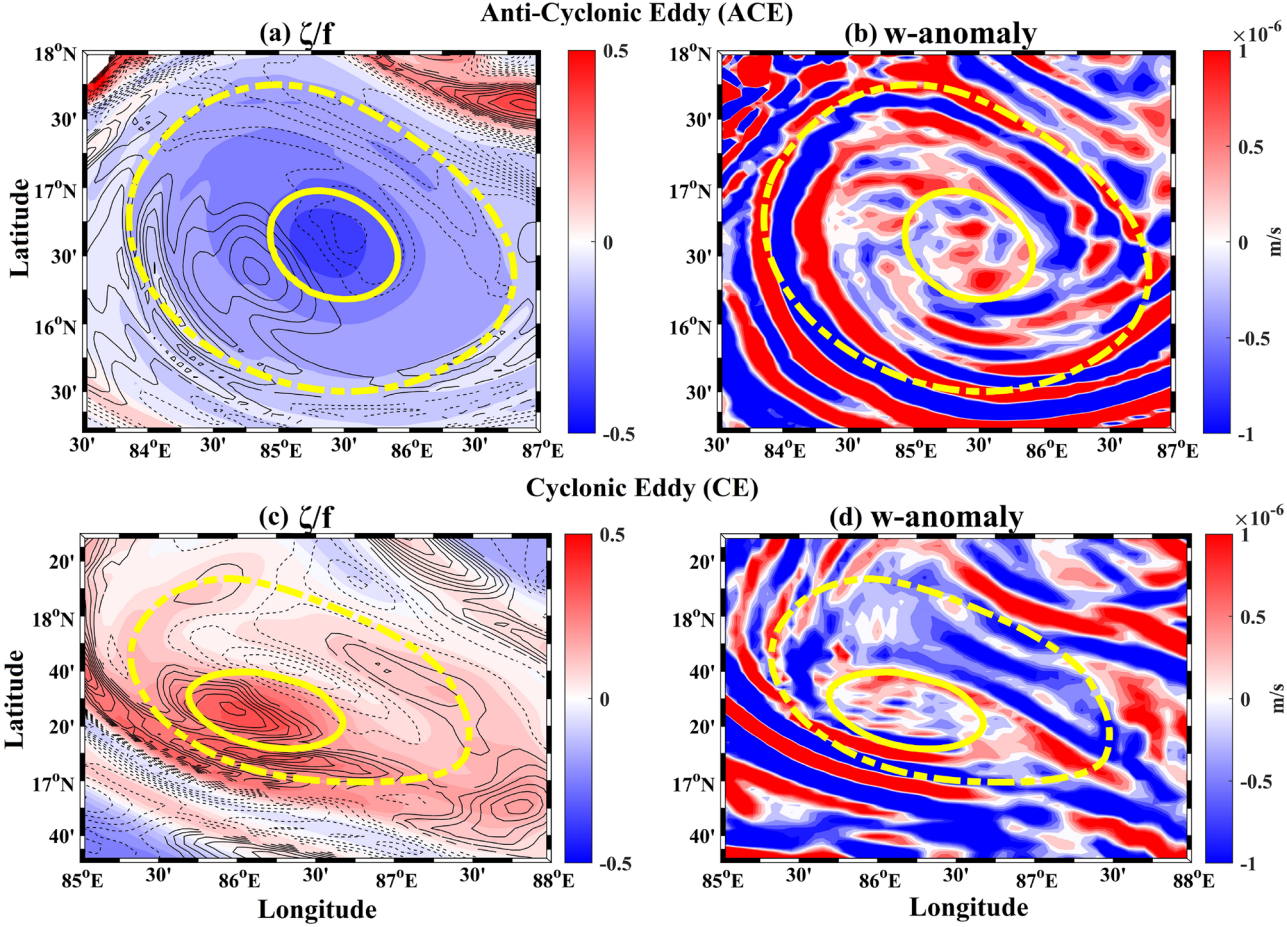
(**a**) Relative vorticity scaled by the Coriolis parameter ($\zeta /f$) with black contours (solid for positive; dashed for negative) representing $\zeta /f$ anomaly for the first day of integration, and (**b**) Vertical velocity (w) anomaly for the first day of integration, for the Anti-Cyclonic Eddy (ACE). (**c**) and (**d**) are the same as (**a**) and (**b**) but for the Cyclonic Eddy (CE). The dashed and solid yellow lines represent the boundaries of the VG eddies and the coherent cores respectively.

The results of the advection of the particles seeded at different vertical levels inside the anti-cyclonic eddy and cyclonic eddy are shown in Fig. 4a and b and in Supplementary Figures 2 and 3, respectively. Though the particles were seeded at different 2-D planes, they were advected using the complete 3D velocity fields resulting in 3D trajectories of the particles. Figure 4a and b show the 2D horizontal projections of the vertical displacement of these 3D particle trajectories, i.e. the difference between the vertical depth of each particle after advection over the integration period (coherence period) and their respective starting depth ($d_{s}$). The main observation here is that different particles, starting from the same vertical level, are going up (upwell) or down (downwell) simultaneously inside the same eddy. Moreover, the anti-cyclonic eddy shows downwelling around the center region with alternating spiral branches surrounding it (Fig. 4a and Supplementary Figure 2). This phenomenon is opposite in sense for the cyclonic eddy with some marked differences in its spiralling patterns and the strength of upwelling/downwelling. The cyclonic eddy also shows a dipole nature of upwelling (southeast section) and downwelling (northwest section) below a certain depth (Fig. 4b and Supplementary Figure 3). We attribute the realization of spiral bands in the 2D horizontal projections to the influence of the VRWs on the particle trajectories. The VRWs manifest as small-scale spiral bands of potential vorticity or vertical velocity^15,16,18–20^, the signatures of which can be seen in the Fig. 5. It should be noted that the VRW signature in the cyclonic eddy is spatially less organized than in the case of the anti-cyclonic eddy. These observations then pose the question about the exact nature of the dynamics occurring inside the coherent eddies and its relation to the existing notion of eddy-induced upwelling and downwelling.

### Continuity analysis: volume conservation of the eddies

In an effort to answer the question posed in the last section, we checked for fluid continuity inside the coherent eddies while advecting them. Since the coherent eddy boundaries are material surfaces, they do not permit any advective material flux through them. On top of that, considering ocean water to be incompressible, the entire exercise of checking for fluid continuity inside the coherent eddies reduces down to the problem of checking for volume conservation of the eddies. Both the anti-cyclonic eddy and the cyclonic eddy show increasing relative volume fraction with advection (Fig. 4c and d). A major part of this might be due to the numerical method used to calculate the volume of the eddies (see Methods) and the rest, though very small, is due to gradual loss of coherence as explained in the previous section. However, it should be noted that this increase in relative volume fraction is very small (maximum values of about 10% and 5% for the anti-cyclonic eddy and the cyclonic eddy respectively) to warrant any material loss or gain by the coherent eddies. Hence, the results of this analysis strongly suggest that the material integrity is maintained throughout the coherence period. Moreover, we also observed that on advection the coherent eddies do stretch or shrink vertically, albeit by a small amount. They deform in the lateral direction with stretching (shrinking) in one direction compensated by a shrinking (stretching) in the other direction.

### Analysis of streamlines and particle trajectories

**Figure 6:**
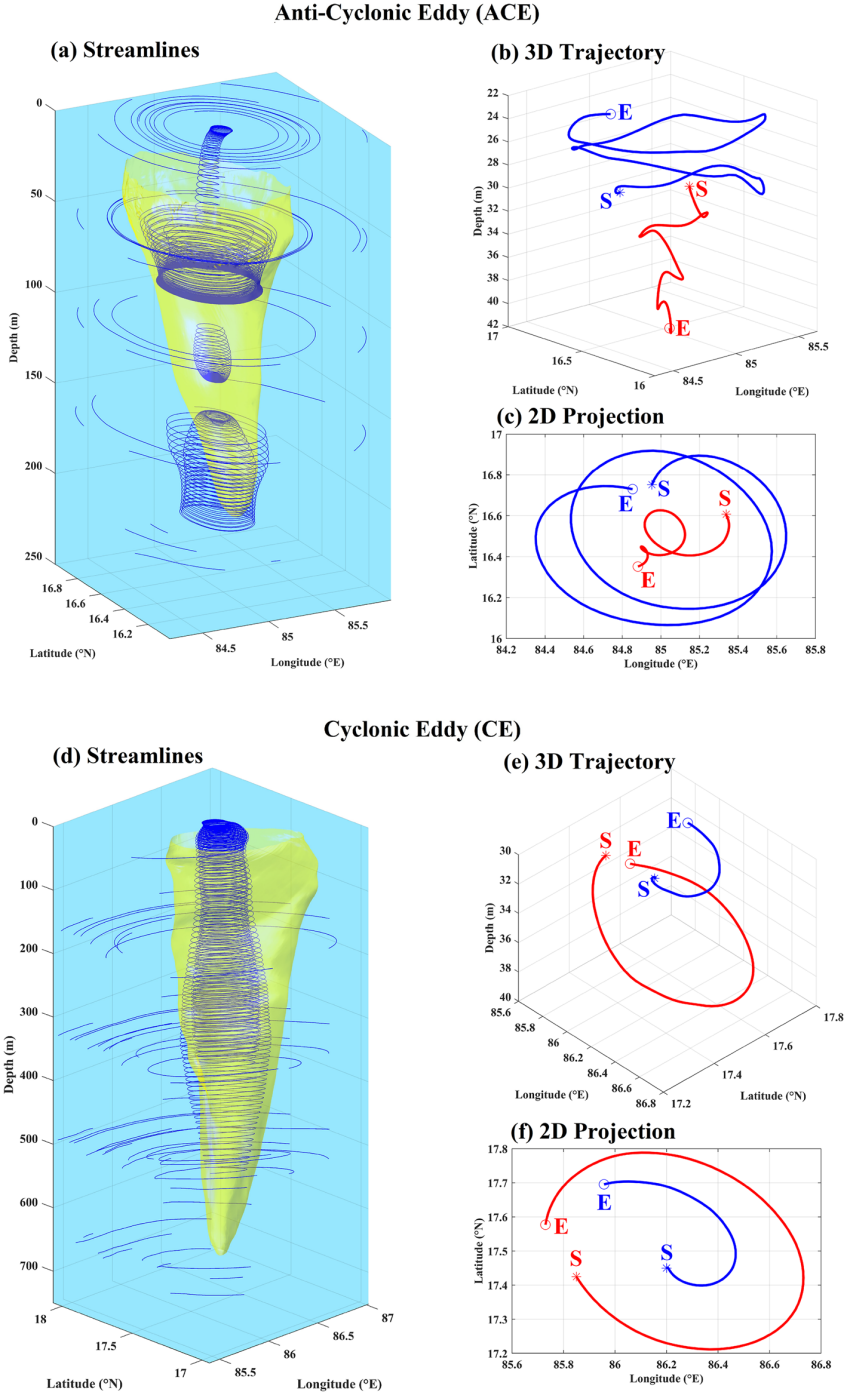
(**a**) Three-dimensional streamlines (blue solid curves) inside and in the vicinity of the coherent eddy structures (yellow; translucent), (**b**) 3D particle trajectory, and (**c**) its 2D projection, wherein blue and red curves represent particle moving up and down, respectively for the Anti-Cyclonic Eddy (ACE). (**d**–**f**) are similar to (**a**–**c**) but for the Cyclonic Eddy (CE). Note that for the ACE the blue and red particles started from close to the boundary and center respectively, vice-versa for the CE. The starting depth for the particle trajectories is 31 m in all the cases. The markers S (*) and E (o) are the starting and ending positions.

Having established the material integrity and the upwelling/downwelling characteristics of the coherent eddies, the final step in formulating the dynamical picture of the interior of coherent eddy was to examine the paths taken by the fluid parcels inside the coherent eddies. This can be achieved first by the streamlines which can give insight about the nature of the flow, and second by observing the actual particle trajectories. Figure 6a and d show select sets of streamlines inside the coherent core and just outside of the coherent core boundary. The streamlines in the interior form long and continuous tapered helices (with the axes aligned with the vertical), but the ones outside the eddies fail to complete full revolutions. This is to be expected as LCSs (the coherent eddy boundaries in this case) separate distinct dynamical regimes of the flow. The idea of helical paths is also corroborated by the actual three-dimensional particle trajectories (Fig. 6b and e) which appear to be spirals in their respective 2D projections (Fig. 6c and f). In the anti-cyclonic eddy (Fig. 6b–c), the particle starting from the periphery and the center show upwelling and downwelling respectively, which is opposite in sense for the cyclonic eddy (Fig. 6e–f).

### Conceptual picture of the interior dynamics of the coherent eddies

Based on the analyses presented above, the following can be concluded about the characteristics and dynamical behavior of the coherent eddies:

- Characteristics﻿ – the three-dimensional structure of the Lagrangian coherent eddies resembles a distorted conical shape. The inverted cone is broader on the surface and tapers with depth (Fig. 3).
- Evolution of the eddy structure – the structures identified remain coherent during the integration period and act as an advective barrier between the inside and outside flow (Supplementary Video 1 and 2).
- Lagrangian particle advection – both upwelling and downwelling occur simultaneously in each of the eddies. The anti-cyclonic eddy shows downwelling in the center region with alternating spiral branches surrounding it. The cyclonic eddy shows an opposite pattern with upwelling in the center, however with marked differences in its realization, compared to the anti-cyclonic eddy, due to factors: (i) the vorticity extremum is off from the center of the circulation pattern (Fig. 1), (ii) VG-based eddy and coherent core axes are misaligned (Fig. 2), and (iii) less organized VRW signature (Fig. 5). The spiral-like patterns exhibited in the 2D horizontal projections of the vertical displacements (at different depths) are the manifestations of VRWs (Figs. 4a–b, 5).
- Continuity – the volume remains conserved (within the limits of numerical accuracy) during the integration period, thereby validating continuity. This suggests that there is no net mass transport into or out of the coherent eddies. Note that the eddies gradually lose material coherence through filamentation which becomes a gateway for mass exchange with the ambient flow (Fig. 4c–d).
- Streamlines and particle trajectories – the 3D streamlines suggest a strong helical nature of the flow in the vertical direction inside the coherent core compared to its outside vicinity (Fig. 6a and d), which is also evident in the particle trajectories (Fig. 6b–c and e–f).

**Figure 7:**
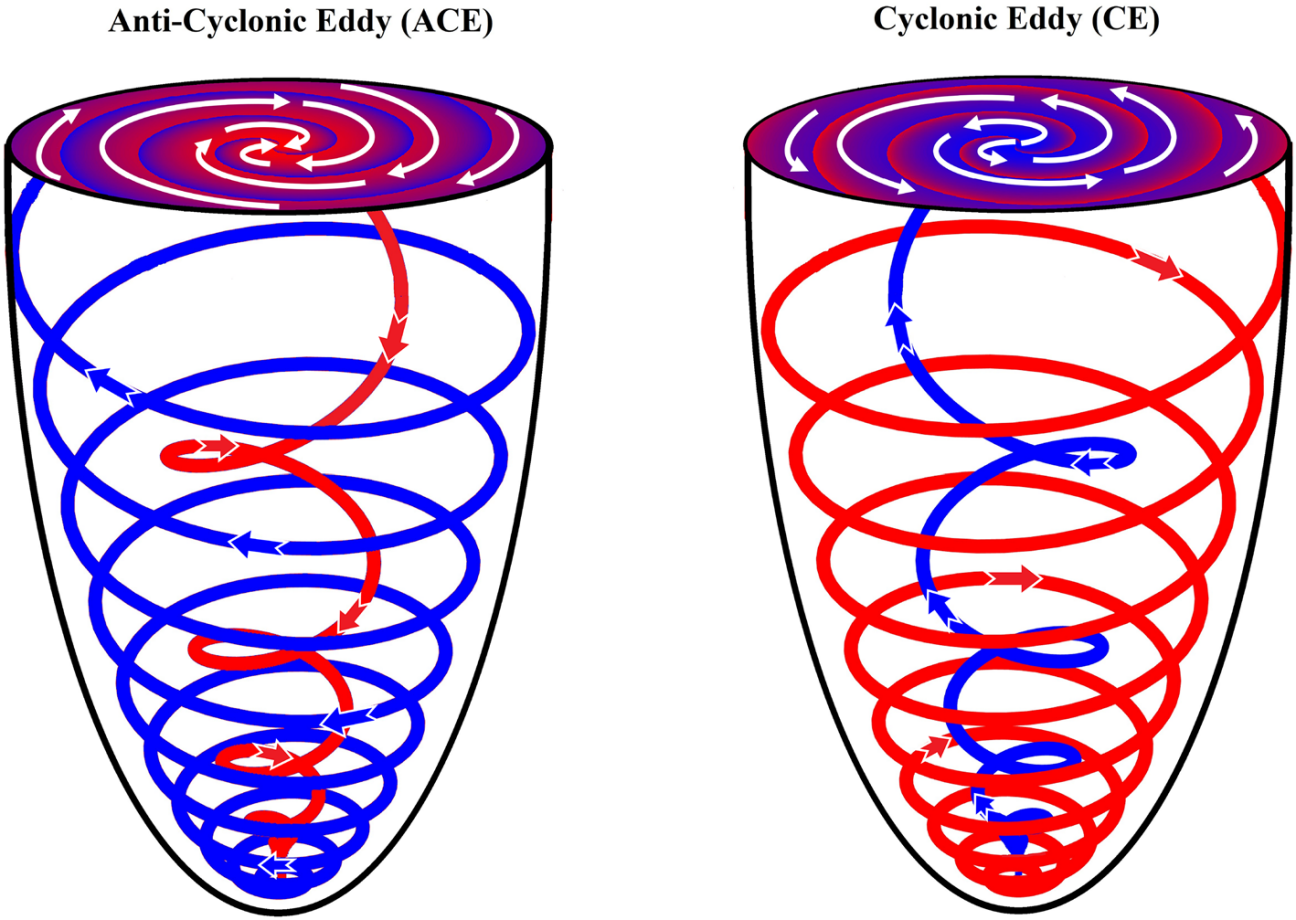
Conceptual picture of the circulation in the interior of the Anti-cyclonic and Cyclonic Eddy. The solid blue (red) lines show the ideal trajectories of the upwelling (downwelling) fluid parcels. The white arrows show the direction of flow on the upper part of the eddies.

These observations can be combined to formulate a consistent dynamical picture of the circulations for the interior of these coherent eddies. For the anti-cyclonic eddy, fluid parcels near the top (bottom) of the structure move helically downward (upward) in 3D around the center (boundaries), with 2D horizontal projections forming spirals due to the influence VRWs (Fig. 6 and Supplementary Video 3–4). The 3D circulation pattern in the cyclonic eddy is similarly helico-spiral, and in the opposite sense of direction (Supplementary Video 5–6). Thus, the overall dynamics inside the coherent core is that of a helico-spiral recirculation as long as the eddies remain coherent (Fig. 7).

## Discussion

Several previous studies have consistently highlighted the importance of fully characterizing the three-dimensional dynamics of eddies and their associated coherent cores^35,40,44,60^. In this study, we have identified the three-dimensional structures of Eulerian eddies derived using the Vector Geometry method^26^ and analyzed their associated coherent cores using the 3D LAVD approach^32^. Our investigation into the dynamical behavior of these coherent cores involved Lagrangian particle advection. Our findings reveal the following:

- The coherent cores are nestled inside larger Eulerian VG-based eddies (Fig. 2). These coherent eddy cores exhibit a conical shape, albeit somewhat distorted, with a broader surface that tapers towards the bottom (Fig. 3).
- These coherent cores maintain their material integrity despite some filamentation upon advection (Supplementary Video 1 and 2). As a consequence, the volume of these coherent cores remains conserved with advection (Fig. 4c–d).
- On performing Lagrangian advection of particles seeded inside the eddy, it was found that both upwelling and downwelling happen simultaneously inside the coherent eddies, forming spiral band structures in the horizontal plane (Fig. 4a–b); the horizontal spiral bands result from the interactions with Vortex Rossby Waves (Fig. 5).
- The 3D streamlines within these coherent cores suggests a strong helical nature of the flow in the vertical direction compared to their surrounding (Fig. 6), which is also evident in the numerical particle trajectories (Fig. 6b–c and e–f).

In summary, our findings collectively paint a three-dimensional picture of the interior circulation within the coherent cores of eddies, which is of a persistent helico-spiral recirculation without any loss or gain of material throughout the period of coherence (as illustrated in Fig. 7). These results open up several avenues for future research.

The subjects of this study were the two prominent mesoscale eddies (an anti-cyclonic eddy and a cyclonic eddy) that form during the eastward flexion of the spring-time WBC in the BoB. Future research could include quantifying the spiralling behavior in eddies due to VRWs, the asymmetry between anti-cyclonic and cyclonic eddies, the screw-like behavior exhibited by the coherent cores of eddies, and on how to reconcile the existing ideas of eddy-induced upwelling and downwelling^61^ with the dynamical aspects of the coherent cores using finer-resolution models^17^ and observations^62^. These coherent eddy cores in the ocean might act as pathways for mass and energy exchange between the atmosphere and the ocean, potentially affect climate variability^13^, result in anomalous air-sea interaction during monsoon^12^, impact biological productivity^63^, and influence cyclone-eddy interaction^9^.

## Methods

### Model description

This study uses ocean currents obtained from ROMS simulation to identify Lagrangian coherent eddies using the LAVD approach. ROMS is a three-dimensional, free surface, primitive equation ocean model based on the hydrostatic balance and the Boussinesq approximation, and follows bathymetry-based vertical sigma coordinate system^53,54^. First, the ROMS was configured for the Indian Ocean domain (34° E–118° E and 26° S–29° N) with a horizontal resolution of 25 km × 25 km and 42 sigma levels. It was integrated for the period 1979–2018 using the monthly forcings of net heat-flux, air temperature, and wind stresses taken from the TropFlux datasets^64^. The initial and boundary conditions for temperature and salinity were obtained from the World Ocean Atlas 2018 datasets. Secondly, a high-resolution configuration was used for the western Bay of Bengal region (79° E–90° E and 10° N–23° N) with a 5 km × 5 km horizontal resolution and with 42 sigma levels. The surface stretching parameter ($\theta _s$) and bottom stretching parameter ($\theta _b$) were taken as 7.0 and 0.58, respectively^65^. The initial and boundary conditions for this nested domain (Fig. 1a) was taken from the lower resolution Indian Ocean model. Ninety-days of daily simulated fields from the 5 km, 42 vertical level simulation were then used for analysis in this study and the model validations are done on similar temporal and spatial scales as that of the observations.

### Vector geometry based Eulerian eddy detection

The VG-based eddy detection algorithm is an Eulerian method to identify eddy centres, boundaries, and tracks from the velocity vectors of the flow field. The eddy centres are the grid points that satisfy the following criteria:

- The zonal component of current has to reverse sign across the centre with magnitude increasing away from the centre,
- The meridional component of current has to reverse sign across the centre with magnitude increasing away from the centre; the sense of rotation should be the same as that of the zonal component,
- The magnitude of velocity has a local minimum around the centre, and
- Around the eddy center, the directions of the velocity vectors have to change with a constant sense of rotation and the directions of two neighboring velocity vectors have to lay within the same or two adjacent quadrants.

Based on these criteria, two parameters *a* and *b* are defined that are dependent on the spatial resolution of the current data. The values of $a = 4$ and $b = 3$ were found to work optimally with the data used in this work. Once the eddy centres are identified, the algorithm determines the eddy boundaries by the closed contours of the stream function field about the eddy centres. Details about the VG-based algorithm can be found in Nencioli *et al.*^26^.

Note that the VG-based algorithm is designed to identify 2D eddy boundaries on horizontal planes. So to obtain the three-dimensional structure of the VG-based eddies, we identified their 2D structures on different horizontal planes at different depths and reconstructed the 3D structure using linear interpolation.

### Lagrangian Averaged Vorticity Deviation (LAVD)

The LAVD is a Lagrangian method to identify both two- and three-dimensional coherent vortices. It should be noted that the Lagrangian methods are finite-time by construction where the integration period depends upon how long a structure persists w.r.t. the ambient flow.

The LAVD (equation 1), defined as the trajectory integral of the normed deviation of the vorticity from its spatial mean, is an objective quantity whose outermost convex isosurfaces correspond to the boundaries of coherent eddies^32^:

1 $$\begin{aligned} \text {LAVD}(\mathbf {x_{0}}) {:}{=}\int _{t_{0}}^{t} \mid \varvec{\omega }({\textbf{x}}(s;\mathbf {x_{0}}),s) - \bar{\varvec{\omega }}(s)\mid \text {ds} \end{aligned}$$

In this equation, $\mathbf {x_{0}} \in U(t)$ are the particle positions at initial time $t_{0}$ with $U(t) \subset {\mathbb {R}}^{3}$ being the spatial domain on which the velocity field ${\textbf{v}}({\textbf{x}}, t)$ is defined, *t* is the final time up to which the particle trajectories are integrated (referred to as the integration period), $\omega$ represents vorticity fields, and ${\bar{\omega }}$ represents the spatial mean of vorticity which is calculated as

2 $$\begin{aligned} \bar{\varvec{\omega }}(t) = \dfrac{\int _{U(t)^{}} \varvec{\omega }(\textbf{x,t}) \, \text {d}V}{\text {vol}(U(t))}, \end{aligned}$$

where $\text {vol}(\cdot )$ denotes the volume for three-dimensional flows, and $\text {d}V$ represents volume elements in *U*(*t*).

The numerical implementation of LAVD used in this study (available at https://github.com/haller-group/Lagrangian-Averaged-Vorticity-Deviation-LAVD.git) works by integrating the trajectories of Lagrangian particles, released from an uniform grid with resolutions *dx*, *dy*, and *dz* in the *x*-, *y*-, and *z*-directions respectively, from initial time $t_{0}$ to a final time *t*. It should be noted that we performed the LAVD computations below 30 m from the sea-surface in order to avoid boundary-effects^32^. The integration was performed using the explicit Runge-Kutta (4,5) scheme and the discrete velocity data was interpolated using a linear gridded-interpolation in both space and time. Once the LAVD fields are computed, the surface structures of the coherent eddies are identified from it using a contour-extraction algorithm. This algorithm has four parameters — (i) *BaseLayerIndex*, (ii) *Nct*, (iii) *MinLength*, and (iv) *DeficiencyThresh*; the details of these parameters can be found in the aforementioned link. The particular values of these parameters used in the current study are summarized in Table 1. Finally, the three-dimensional structure of the coherent eddy is delineated by an isosurface generation algorithm based on the values of these parameters. The readers are referred to Haller et al. (2016)^32^ for a detailed discussion on LAVD and its numerical implementations.

**Table 1:** Parameters used in LAVD computation for both the Anti-Cyclonic Eddy (ACE) and Cyclonic Eddy (CE).

	Integration period		Days	dx (km)	dy (km)	dz (m)	dt (days)	BaseLayerIndex	Nct	MinLength	DeficiencyThresh
	From	To									
ACE	26-Mar-17	09-Apr-17	15	1.0	1.0	5.0	0.067	1	25	0.40	0.20
CE	28-Apr-17	06-May-17	9	1.0	1.0	5.0	0.067	1	25	1.00	0.20

### Lagrangian particle advection

The three-dimensional structure of the coherent eddies was broken down into different vertical levels. A grid of 100 × 100 neutrally-buoyant particles were generated on a rectangular grid defined by the longitudinal and latitudinal extents of the coherent eddy boundary at those particular vertical levels. Then, the particles falling inside the eddy boundary were selected for advection. Finally, the advection was performed using the explicit Runge-Kutta (4,5) scheme and the discrete velocity data was interpolated using a linear gridded-interpolation in both space and time.

The volumes of the coherent eddy structures were computed by fitting $\alpha$-shapes to the eddy boundaries. Fitting convex hulls was avoided as it was unable to capture small-scale deformations and filamentations in the eddy boundaries during advection, and hence often under or overestimated the volumes.

### Streamline computation

Streamlines are curves that are everywhere tangent to the direction of velocity field at a given instant of time^66^. The three-dimensional streamlines are computed as:

3 $$\begin{aligned} {\textbf{u}}({\textbf{x}},t) \times d{\textbf{s}} = 0 \end{aligned}$$

where, ${\textbf{u}}({\textbf{x}},t)$ is the 3D spatial velocity field at a particular instant of time (*t*) and $d{\textbf{s}}$ is an infinitesimal length element in three-dimensional space (${\textbf{x}}$).

## Supplementary Information

Supplementary Information 1.

Supplementary Information 2.

Supplementary Information 3.

Supplementary Information 4.

Supplementary Information 5.

Supplementary Information 6.

Supplementary Information 7.

## Data Availability

Monthly forcings of net heat-flux, air temperature, and wind stress datasets have been obtained from the Indian National Centre for Ocean Information Services (INCOIS) TropFlux (https://incois.gov.in/tropflux/DataHome.jsp; registration required). World Ocean Atlas 2018 datasets are available at https://www.ncei.noaa.gov/access/world-ocean-atlas-2018/. Daily ocean surface-current is taken from Indian Space Research Organization (ISRO)^57^ (https://www.mosdac.gov.in/opendata/ocean_surface_current; registration required). Copernicus Marine Service (CMEMS) provides SLA (AVISO) data https://doi.org/10.48670/moi-00148. The numerical implementation of LAVD can be obtained from https://github.com/haller-group/Lagrangian-Averaged-Vorticity-Deviation-LAVD/tree/master/3D). All the links are accessible on 14th November, 2023. The ROMS model simulation outputs can be made available on request.

## References

[1] Ducet, N., Le Traon, P.-Y. & Reverdin, G. Global high-resolution mapping of ocean circulation from topex/poseidon and ers-1 and-2. *J. Geophys. Res. Oceans* **105**, 19477–19498 (2000).

[2] McWilliams, J. C. The nature and consequences of oceanic eddies. *Ocean Model. Eddy. Regime* **177**, 5–15. 10.1029/177GM03 (2008).

[3] Robinson, A. R. *Eddies in marine science* (Springer, 2012).

[4] Ferrari, R. & Wunsch, C. Ocean circulation kinetic energy: Reservoirs, sources, and sinks. *Annu. Rev. Fluid Mech.* **41**, 253–282. 10.1146/annurev.fluid.40.111406.102139 (2009).

[5] Vallis, G. K. *Atmospheric and oceanic fluid dynamics* (Cambridge University Press, 2017).

[6] Gangopadhyay, A. *Introduction to Ocean Circulation and Modeling* (CRC Press, 2022).

[7] Zhang, Z., Wang, W. & Qiu, B. Oceanic mass transport by mesoscale eddies. *Science* **345**, 322–324. 10.1126/science.1252418 (2014). 25035491 10.1126/science.1252418

[8] Ali, M., Jagadeesh, P. V. & Jain, S. Effects of eddies on bay of bengal cyclone intensity. *EOS Trans. Am. Geophys. Union* **88**, 93–95 (2007).

[9] Jangir, B., Swain, D. & Ghose, S. Influence of eddies and tropical cyclone heat potential on intensity changes of tropical cyclones in the north indian ocean. *Adv. Space Res.* **68**, 773–786 (2021).

[10] Sil, S., Gangopadhyay, A., Gawarkiewicz, G. & Pramanik, S. Shifting seasonality of cyclones and western boundary current interactions in bay of bengal as observed during amphan and fani. *Sci. Rep.* **11**, 22052 (2021). 34764378 10.1038/s41598-021-01607-6 PMC8586239

[11] Gupta, H., Sil, S., Gangopadhyay, A. & Gawarkiewicz, G. Observed surface and subsurface marine heat waves in the bay of bengal from in-situ and high-resolution satellite data. *Clim. Dyn.* 1–19 (2023).

[12] Gulakaram, V. S., Vissa, N. K. & Bhaskaran, P. K. Role of mesoscale eddies on atmospheric convection during summer monsoon season over the bay of bengal: A case study. *J. Ocean Eng. Sci.* **3**, 343–354 (2018).

[13] Kirtman, B. P., Perlin, N. & Siqueira, L. Ocean eddies and climate predictability. *Chaos Interdiscip. J. Nonlinear Sci.* **27** (2017). 10.1063/1.4990034 29289056

[14] Cushman-Roisin, B. & Beckers, J.-M. *Introduction to geophysical fluid dynamics: Physical and numerical aspects* (Academic press, 2011).

[15] Koszalka, I., Bracco, A., McWilliams, J. C. & Provenzale, A. Dynamics of wind-forced coherent anticyclones in the open ocean. *J. Geophys. Res. Oceans* **114** (2009).

[16] Nardelli, B. B. Vortex waves and vertical motion in a mesoscale cyclonic eddy. *J. Geophys. Res. Oceans* **118**, 5609–5624 (2013).

[17] Zhong, Y. *et al.* Observed and simulated submesoscale vertical pump of an anticyclonic eddy in the south china sea. *Sci. Rep.* **7**, 44011 (2017). 28276467 10.1038/srep44011 PMC5343664

[18] Montgomery, M. T. & Kallenbach, R. J. A theory for vortex rossby-waves and its application to spiral bands and intensity changes in hurricanes. *Q. J. R. Meteorol. Soc.* **123**, 435–465 (1997).

[19] McWilliams, J. C., Graves, L. P. & Montgomery, M. T. A formal theory for vortex rossby waves and vortex evolution. *Geophys. Astrophys. Fluid Dyn.* **97**, 275–309 (2003).

[20] Graves, L. P., McWilliams, J. C. & Montgomery, M. T. Vortex evolution due to straining: A mechanism for dominance of strong, interior anticyclones. *Geophys. Astrophys. Fluid Dyn.* **100**, 151–183 (2006).

[21] Morel, Y. & Thomas, L. N. Ekman drift and vortical structures. *Ocean Model.* **27**, 185–197 (2009).

[22] Mahadevan, A. *et al.* Coherent pathways for vertical transport from the surface ocean to interior. *Bull. Am. Meteor. Soc.* **101**, E1996–E2004 (2020).

[23] D’Asaro, E. A. *et al.* Advances in observing and understanding small-scale open ocean circulation during the gulf of mexico research initiative era. *Front. Mar. Sci.* **7**, 349 (2020).

[24] Zhang, Z. & Qiu, B. Surface chlorophyll enhancement in mesoscale eddies by submesoscale spiral bands. *Geophys. Res. Lett.* **47**, e2020GL088820 (2020).

[25] Chelton, D. B., Schlax, M. G., Samelson, R. M. & de Szoeke, R. A. Global observations of large oceanic eddies. *Geophys. Res. Lett.* **34**, 10.1029/2007GL030812 (2007).

[26] Nencioli, F., Dong, C., Dickey, T., Washburn, L. & McWilliams, J. C. A vector geometry-based eddy detection algorithm and its application to a high-resolution numerical model product and high-frequency radar surface velocities in the southern california bight. *J. Atmos. Oceanic Tech.* **27**, 564–579. 10.1175/2009JTECHO725.1 (2010).

[27] Hunt, J. C., Wray, A. A. & Moin, P. Eddies, streams, and convergence zones in turbulent flows. *Studying turbulence using numerical simulation databases, 2. Proceedings of the 1988 summer program* (1988).

[28] Chong, M. S., Perry, A. E. & Cantwell, B. J. A general classification of three-dimensional flow fields. *Phys. Fluids A* **2**, 765–777. 10.1063/1.857730 (1990).

[29] Hua, B. & Klein, P. An exact criterion for the stirring properties of nearly two-dimensional turbulence. *Physica D* **113**, 98–110. 10.1016/S0167-2789(97)00143-7 (1998).

[30] Jeong, J. & Hussain, F. On the identification of a vortex. *J. Fluid Mech.* **285**, 69–94. 10.1017/S0022112095000462 (1995).

[31] Beron-Vera, F. J., Wang, Y., Olascoaga, M. J., Goni, G. J. & Haller, G. Objective detection of oceanic eddies and the agulhas leakage. *J. Phys. Oceanogr.* **43**, 1426–1438. 10.1175/JPO-D-12-0171.1 (2013).

[32] Haller, G., Hadjighasem, A., Farazmand, M. & Huhn, F. Defining coherent vortices objectively from the vorticity. *J. Fluid Mech.* **795**, 136–173. 10.1017/jfm.2016.151 (2016).

[33] Haller, G. An objective definition of a vortex. *J. Fluid Mech.* **525**, 1–26. 10.1017/S0022112004002526 (2005).

[34] Haller, G. Lagrangian coherent structures. *Annu. Rev. Fluid Mech.* **47**, 137–162. 10.1146/annurev-fluid-010313-141322 (2015).

[35] Abernathey, R. & Haller, G. Transport by lagrangian vortices in the eastern pacific. *J. Phys. Oceanogr.* **48**, 667–685. 10.1175/JPO-D-17-0102.1 (2018).

[36] Denes, M. C., Froyland, G. & Keating, S. R. Persistence and material coherence of a mesoscale ocean eddy. *Phys. Rev. Fluids* **7**, 034501. 10.1103/PhysRevFluids.7.034501 (2022).

[37] Flierl, G. Isolated eddy models in geophysics. *Annu. Rev. Fluid Mech.* **19**, 493–530. 10.1146/annurev.fl.19.010187.002425 (1987).

[38] Provenzale, A. Transport by coherent barotropic vortices. *Annu. Rev. Fluid Mech.* **31**, 55–93. 10.1146/annurev.fluid.31.1.55 (1999).

[39] Haller, G. & Beron-Vera, F. J. Coherent lagrangian vortices: The black holes of turbulence. *J. Fluid Mech.* **731**, R4. 10.1017/jfm.2013.391 (2013).

[40] Liu, T., Abernathey, R., Sinha, A. & Chen, D. Quantifying eulerian eddy leakiness in an idealized model. *J. Geophys. Res. Oceans* **124**, 8869–8886. 10.1029/2019JC015576 (2019).

[41] Cetina-Heredia, P., Roughan, M., van Sebille, E., Keating, S. & Brassington, G. B. Retention and leakage of water by mesoscale eddies in the east australian current system. *J. Geophys. Res. Oceans* **124**, 2485–2500. 10.1029/2018JC014482 (2019).

[42] Beron-Vera, F. J., Olascoaga, M. J. & Goni, G. Oceanic mesoscale eddies as revealed by lagrangian coherent structures. *Geophys. Res. Lett.* **35**, 10.1029/2008GL033957 (2008).

[43] Bettencourt, J. H., López, C. & Hernández-García, E. Oceanic three-dimensional lagrangian coherent structures: A study of a mesoscale eddy in the benguela upwelling region. *Ocean Model.* **51**, 73–83. 10.1016/j.ocemod.2012.04.004 (2012).

[44] Tarshish, N. *et al.* Identifying lagrangian coherent vortices in a mesoscale ocean model. *Ocean Model.* **130**, 15–28. 10.1016/j.ocemod.2018.07.001 (2018).

[45] El Aouni, A., Daoudi, K., Yahia, H., Maji, S. K. & Minaoui, K. Defining lagrangian coherent vortices from their trajectories. *Phys. Fluids* **32**, 016602. 10.1063/1.5138899 (2020).

[46] Blazevski, D. & Haller, G. Hyperbolic and elliptic transport barriers in three-dimensional unsteady flows. *Physica D* **273**, 46–62 (2014).

[47] Pierrehumbert, R. T. & Yang, H. Global chaotic mixing on isentropic surfaces. *J. Atmos. Sci.* **50**, 2462–2480 (1993).

[48] Lapeyre, G. Characterization of finite-time lyapunov exponents and vectors in two-dimensional turbulence. *Chaos Interdiscip. J. Nonlinear Sci.* **12**, 688–698 (2002). 10.1063/1.1499395 12779597

[49] d’Ovidio, F., Fernández, V., Hernández-García, E. & López, C. Mixing structures in the mediterranean sea from finite-size lyapunov exponents. *Geophys. Res. Lett.* **31** (2004).

[50] Cencini, M. & Vulpiani, A. Finite size lyapunov exponent: Review on applications. *J. Phys. A Math. Theor.* **46**, 254019. 10.1088/1751-8113/46/25/254019 (2013).

[51] Wang, Y., Olascoaga, M. J. & Beron-Vera, F. J. Coherent water transport across the south atlantic. *Geophys. Res. Lett.* **42**, 4072–4079. 10.1002/2015GL064089 (2015).

[52] Xia, Q., Li, G. & Dong, C. Global oceanic mass transport by coherent eddies. *J. Phys. Oceanogr.* **52**, 1111–1132. 10.1175/JPO-D-21-0103.1 (2022).

[53] Penven, P., Debreu, L., Marchesiello, P. & McWilliams, J. C. Evaluation and application of the roms 1-way embedding procedure to the central california upwelling system. *Ocean Model.* **12**, 157–187. 10.1016/j.ocemod.2005.05.002 (2006).

[54] Shchepetkin, A. F. & McWilliams, J. C. The regional oceanic modeling system (roms): A split-explicit, free-surface, topography-following-coordinate oceanic model. *Ocean Model.* **9**, 347–404. 10.1016/j.ocemod.2004.08.002 (2005).

[55] Gangopadhyay, A., Bharat Raj, G., Chaudhuri, A. H., Babu, M. & Sengupta, D. On the nature of meandering of the springtime western boundary current in the bay of bengal. *Geophys. Res. Lett.* **40**, 2188–2193, 10.1002/grl.50412 (2013).

[56] Chen, G., Li, Y., Xie, Q. & Wang, D. Origins of eddy kinetic energy in the bay of bengal. *J. Geophys. Res. Oceans* **123**, 2097–2115. 10.1002/2017JC013455 (2018).

[57] Sikhakolli, R. *et al.* Improved determination of Indian Ocean surface currents using satellite data. *Remote Sens. Lett.* **4**, 335–343. 10.1080/2150704X.2012.730643 (2013).

[58] Li, H., Xu, F. & Wang, G. Global mapping of mesoscale eddy vertical tilt. *J. Geophys. Res. Oceans* e2022JC019131, 10.1029/2022JC019131 (2022).

[59] Li, H., Xu, F., Wang, G. & Shi, R. Numerical studies of the tilting of mesoscale eddies: The effects of rotation and stratification. *Deep Sea Res. Part I* **191**, 103945. 10.1016/j.dsr.2022.103945 (2023).

[60] Hopfinger, E. & Van Heijst, G. Vortices in rotating fluids. *Annu. Rev. Fluid Mech.* **25**, 241–289. 10.1146/annurev.fl.25.010193.001325 (1993).

[61] McGillicuddy, D. J. Jr. Mechanisms of physical-biological-biogeochemical interaction at the oceanic mesoscale. *Ann. Rev. Mar. Sci.* **8**, 125–159 (2016). 26359818 10.1146/annurev-marine-010814-015606

[62] Encinas-Bartos, A. P., Aksamit, N. O. & Haller, G. Quasi-objective eddy visualization from sparse drifter data. *Chaos Interdiscip. J. Nonlinear Sci.* **32**, 113143, 10.1063/5.0099859 (2022). 10.1063/5.0099859 36456328

[63] McGillicuddy, D. J. Jr. *et al.* Eddy/wind interactions stimulate extraordinary mid-ocean plankton blooms. *Science* **316**, 1021–1026. 10.1126/science.1136256 (2007). 17510363 10.1126/science.1136256

[64] Praveen Kumar, B., Vialard, J., Lengaigne, M., Murty, V. & Mcphaden, M. J. Tropflux: Air-sea fluxes for the global tropical oceans-description and evaluation. *Clim. Dyn.* **38**, 1521–1543, 10.1007/s00382-011-1115-0 (2012).

[65] Sil, S. & Chakraborty, A. Simulation of east India coastal features and validation with satellite altimetry and drifter climatology. *Int. J. Ocean Clim. Syst.* **2**, 279–289. 10.1260/1759-3131.2.4.279 (2011).

[66] Kundu, P. K., Cohen, I. M. & Dowling, D. R. *Fluid mechanics* (Academic press, 2015).

