# Supplementary material for: On the evidence of helico-spiralling recirculation within coherent cores of eddies using Lagrangian approach
Source: Sci Rep. 2024 May 14;14:11014. doi: 10.1038/s41598-024-61744-6 (PMC11639711; doi:10.1038/s41598-024-61744-6)
Supplement: Supplementary file 1 — Supplementary Information 1. [file 41598_2024_61744_MOESM1_ESM.pdf]

On the Evidence of Helico-Spiralling Recirculation Within Coherent Cores of Eddies Using Lagrangian Approach

Rahul Deogharia[1,*], **Hitesh Gupta**[1], **Sourav Sil**[1], **Avijit Gangopadhyay**[2,1], **and Abhijit Shee**[3]

[1]Ocean Analysis and Modeling Laboratory, School of Earth, Ocean and Climate Sciences, Indian Institute of Technology Bhubaneswar, Khordha, 752050, Odisha, India

[2]School for Marine Science and Technology, University of Massachusetts, Dartmouth, 02747, MA, USA

[3]Centre for Atmospheric and Oceanic Sciences, Indian Institute of Science, Bengaluru, 560012, Karnataka, India

[*]rd16@iitbbs.ac.in

Supplementary Information

Supplementary Text 1 and Figures 1–3.

Supplementary Text 1

Supplementary Video 1:

Left Panel: Time evolution of the surface structure of the anti-cyclonic eddy (red patch) overlaid on the bathymetry (shaded). Vectors represent surface-current fields.

Right Panel: Time evolution of the 3D structure of the anti-cyclonic eddy.

Supplementary Video 2:

Same as Supplementary Video 1 but for the cyclonic eddy.

Supplementary Video 3:

Left Panel: Side view of the time evolution of the particles seeded inside the anti-cyclonic eddy on a selected level during the integration period (Azimuth = 160° and Elevation = 60°). The colorbar represents the instantaneous vertical positions of the particles. The starting depth (d_s) of all the particles is 31m. It can be observed that multiple helical branches of upwelling (z-position $<$ 31m) and downwelling (z-position $>$ 31m) develop surrounding the central region which shows a predominantly downwelling character. Also, note the undulations in these helical branches which are due to the interaction with Vortex Rossby Waves (VRWs).

Right Panel: Same as Left Panel but viewing from the top (Azimuth = 160° and Elevation = 90°). The helical branches, together with the impact of VRWs, combine to form the spiral patterns when observed from the top. These spiral patterns tend to intensify with time.

Supplementary Video 4:

Three-dimensional view of advected particles from different angles on the last day of integration for the anti-cyclonic eddy. The starting depth (d_s) of the particles is 31m. The colorbar indicates the final vertical position of particles and the black dots show the initial grid of seeded particles. The helical branches as seen in Supplementary Video 3 can be discerned better from different angles (rotation along azimuth). About six branches, i.e., three upwelling and three downwelling branches can be seen from different angles. However, this becomes more evident in the view from an elevation of -90° (at the end of the video).

Supplementary Video 5:

Similar to Supplementary Video 3 but for the cyclonic eddy. Left panel: Side View (Azimuth = 160° and Elevation = 40°); Right panel: Top View (Azimuth = 160° and Elevation = 90°).

Supplementary Video 6:

Similar to Supplementary Video 4 but for the cyclonic eddy. Similar to the case of the ACE (Supplementary Video 4), a total of six helical branches are observed with three upwelling and three downwelling branches.

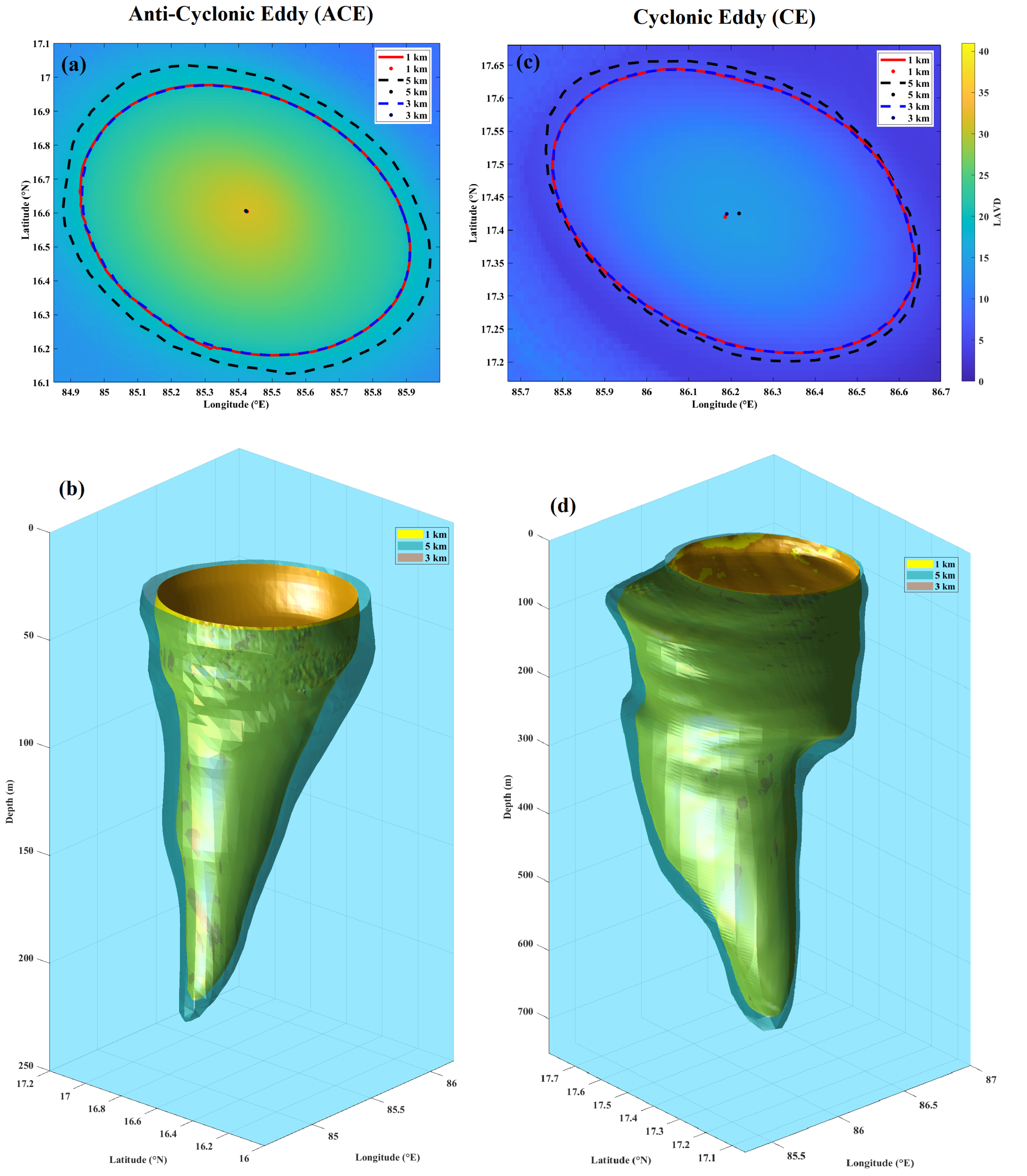

Supplementary Fig. 1. The 2D and 3D structures of the coherent cores as identified through LAVD computations performed at 1km, 3km, and 5km horizontal resolution (with corresponding temporal resolutions of 1/15, 1/10, 1 day) for the (a) and (b) Anti-cyclonic Eddy (ACE), and (c) and (d) Cyclonic Eddy (CE). The 2D eddy boundary from the 1km, 3km, and 5km resolution computations are shown with solid red, blue dashed, and black dashed lines in (a) and (c), and their corresponding 3D structures are shown in yellow, orange, and partially-transparent green in (b) and (d).

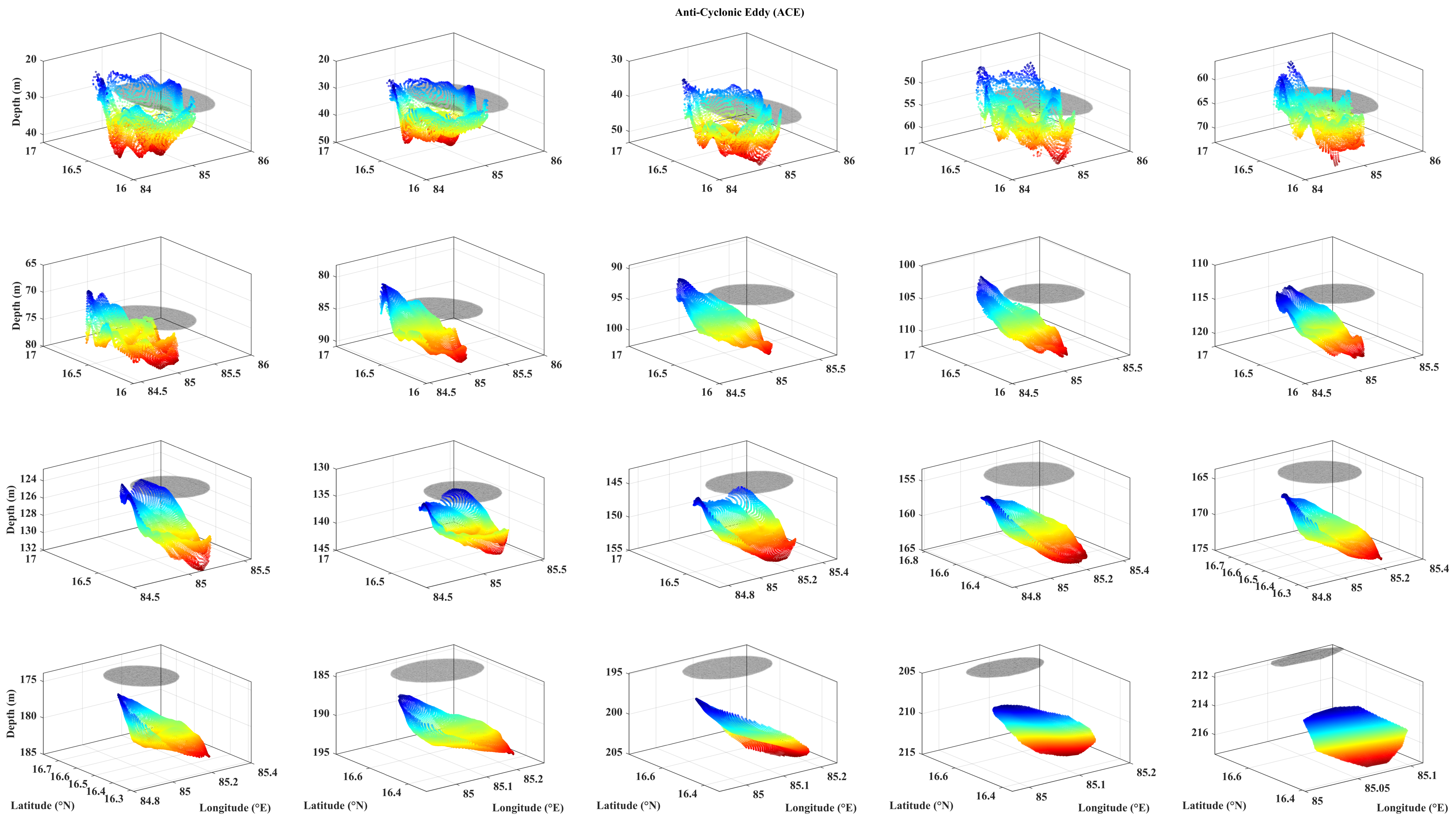

Supplementary Fig. 2. The surfaces of particles advected inside the Anti-Cyclonic Eddy (ACE). The colormap indicates the vertical position of the surfaces at the end of the advection period. The black surface is the original depth level before advection.

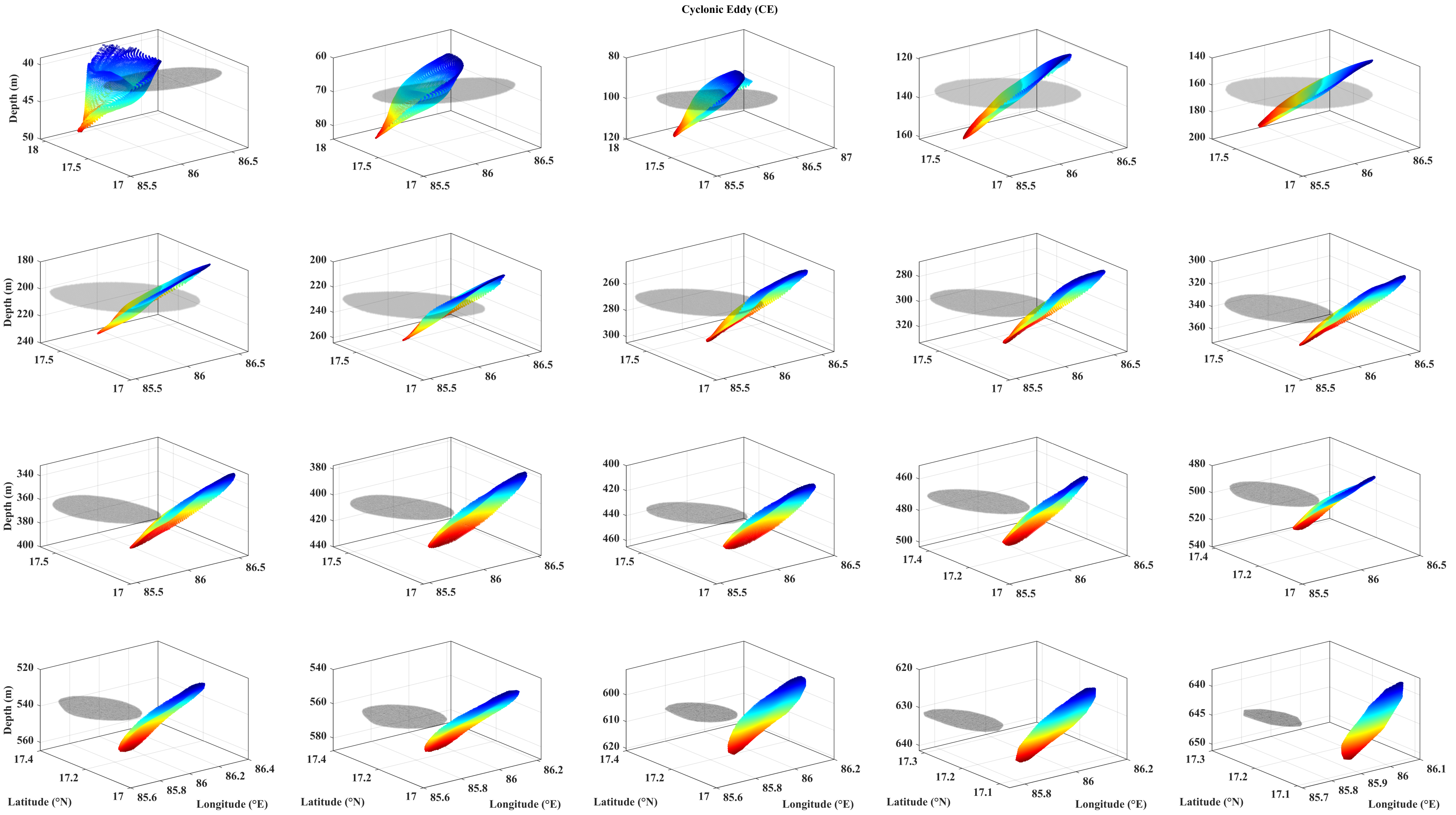

Supplementary Fig. 3. The surfaces of particles advected inside the Cyclonic Eddy (CE). The colormap indicates the vertical position of the surfaces at the end of the advection period. The black surface is the original depth level before advection.
